# Reduction of serum IGF-I levels in patients affected with Monoclonal Gammopathies of undetermined significance or Multiple Myeloma. Comparison with bFGF, VEGF and K-*ras *gene mutation

**DOI:** 10.1186/1756-9966-28-35

**Published:** 2009-03-10

**Authors:** Claudia Greco, Gaetano Vitelli, Giuseppe Vercillo, Rosa Vona, Diana Giannarelli, Isabella Sperduti, Francesco Pisani, Ettore Capoluongo, Maria Concetta Petti, Franco Ameglio

**Affiliations:** 1 Clinical Pathology Service, Polo Oncologico Regina Elena, Rome, Italy; 2 Department of Developmental Neurology and Psychiatry, La Sapienza University, Rome, Italy; 3 Drug Research and Evaluation Department, Section of Cell Aging and Deneration, Istitute Superiore di Sanita, Rome, Italy; 4 Service of Biostatistics, Polo Oncologico Regina Elena, Rome, Italy; 5 Hematology Department, Polo Oncologico Regina Elena, Rome, Italy; 6 Laboratory of Molecular Biology, Institute of Biochemistry & Clinical Biochemistry, Catholic University, Rome, Italy

## Abstract

**Background:**

Serum levels of IGF-I in patients affected with multiple myeloma (MM) have been scarcely studied. The present study is aimed to explore this point comparing 55 healthy subjects, 71 monoclonal gammopaties of uncertain significance (MGUS) and 77 overt MM patients. In the same subjects, basic FGF and VEGF, have been detected. All three mediators were analyzed in function of K-*ras *mutation and melphalan response. Concerning IGF-I, two representative monitoring examples have also been added.

**Methods:**

Cytokine determinations were performed by commercially available ELISA kits, while K12-*ras *mutation was investigated on genomic DNA isolated from bone marrow cell specimens by RFLP-PCR assay.

**Results:**

Significant reductions of IGF-I levels were observed in MGUS and MM as compared with healthy controls. In addition, MM subjects showed significantly decreased serum IGF-I levels than MGUS. Conversely, increasing levels were observed for bFGF and VEGF, molecules significantly correlated. A multivariate analysis corrected for age and gender confirmed the significant difference only for IGF-I values (P = 0.01). K12-*ras *mutation was significantly associated with malignancy, response to therapy and with significantly increased serum bFGF levels.

**Conclusion:**

IGF-I reduction in the transition: Controls→MGUS→MM and changes observed over time suggest that IGF-I should be furtherly studied in future clinical trials as a possible monitoring marker for MM.

## Background

In spite of the progresses recently registered in the therapy of multiple myeloma (MM), the prognosis for patients affected by this disease remains still poor [1]. MM demonstrate a progressive, usually fatal, course with traditional treatments, generally producing only temporary remissions. A 5-year survival for MM patients is about 25% with less than 5% alive subjects after 10 years [1].

Among innovative treatments, antiangiogenic therapy seems to represent a promising approach, whose rationale is based on tumour growth inhibition by starving cancer cells of vital nutrients [2]. Recent evidences indicate that angiogenic processes are increased and are fundamental not only in solid tumours but also in hematologic diseases, including MM, as well [3,4].

Scarce angiogenic activities have been found in monoclonal gammopathy of undetermined significance (MGUS) as compared to the overt malignant forms, where marrow neoangiogenesis parallels tumour progression and correlates with poor prognosis, suggesting an angiogenesis-dependent regulation of disease activity [5-7].

Neoangiogenesis is under the control of various cytokines, that are expressed by neoplastic plasma cells, so that their involvement in MM pathophysiology has been strongly supported by different reports [8]. These modulators include vascular endothelial growth factor (VEGF), hepatocyte growth factor (HGF) and basic fibroblast growth factor (bFGF), that have been extensively investigated in biological samples derived from MM patients. However, data concerning their potential prognostic power as well as their reciprocal interactions are still conflicting [8-10] and remain to be better elucidated.

VEGF is a major regulator of tumour-associated angiogenesis exhibiting various biological activities, including regulation of embryonic stem cell development and local generation of inflammatory cytokines [11]. VEGF gene encodes for at least five isoforms which are anchored to the extracellular matrix through the heparin-binding domains. They are mitogenic to vascular endothelial cells and induce vascular permeabilization [11]. VEGF expression is regulated by several factors including interleukins (IL-1β, IL-6, IL-10), fibroblast growth factor (FGF-4) and insulin-like growth factor1(IGF-1) [12].

bFGF is an 18 to 24 kD polypeptide, mainly produced by cells of mesenchymal origin, which shares a key role of mediator of angiogenesis with VEGF in vitro [13] and in vivo [14]. This molecule is normally bound to heparin and heparan sulphate proteoglycans in the extracellular matrix, particularly in the basement membranes, around vessels and stromal cells. It binds to a family of four distinct, high affinity tyrosine kinase receptors (FGFR-1–4) and stimulates endothelial cell proliferation in vitro [13].

IGF-I is a mitogen and anti-apoptotic cytokine/growth factor/hormone produced by several types of cells (fibroblasts, hepatocytes, chondroblasts ..) [15]. Its potential role as a growth factor for myeloma cells has been deeply analyzed and data of Ge NL et al [16] suggest that IGF-I significantly contributes to the expansion of MM cells in vivo by activation of two distinct pathways: Akt/Bad and MAPK kinase. Moreover, it has been suggested that circulating levels of IGF-I and its inhibitory binding proteins (IGF-BP 1–6) may be associated with an increasing risk of common cancers [17]. The main circulating component of IGF-I is released by the liver under GH control, while locally, different regulatory mechanisms have been reported [18,19]. Free IGF-I (molecules unbound to IGF-BPs) acts through a specific high-affinity IGF-I receptor, but also insulin receptor and IGF-II receptor may be used although with lower affinities [20].

Recent data from the literature seem to support the idea of a functional link existing between the induction of angiogenesis-mediated growth factor expression and gene alterations in tumour development. In particular, c-myc deregulation by PDGF-BB has been demonstrated either in normal [21] or in tumour cells [22]. Moreover, the existence of a relationship between activation of ras oncogenes and regulation of the VEGF/VPF expression has been demonstrated in experimental [23] and clinical [24] studies. In this regard, there are several reasons supporting the fact that *ras *gene represents an interesting case for studying the impact of cancer-associated genetic mutations and tumour angiogenesis. In fact, activated *ras *is capable of triggering several crucial signalling cascades, so altering the expression of some members of *ras*-responsive genes, many of which could be relevant for triggering or contributing to tumour angiogenesis [25]. Although the mechanisms governing the expression of angiogenic cytokines in tumour cell by dominantly acting oncogenes is largely unknown, the regulatory effect of oncogenes on angiogenic mediators has some potentially important therapeutic consequences and needs to be better investigated, especially on hematologic malignancies.

Aim of the present study was to evaluate the serum levels of a panel of three cytokines, such as IGF-I plus two angiogenic factors such as VEGF and bFGF in 148 patients with plasma cell dyscrasias. Seventy-one out of the total were patients affected by MGUS and 77 were patients with MM, these latter receiving treatment with conventional chemotherapy (Melphalan/Prednisone). These two groups of patients were compared with 55 controls represented by healthy human blood donors. In addition, we tried to determine whether the serum levels of these cytokines combined with the K-*ras *gene alterations might allow to select groups of patients with different responsiveness to chemotherapy.

## Methods

### Patients and Controls

One hundred and forty-eight patients affected with plasmacell dyscrasia were consecutively admitted to the Regina Elena Cancer Institute of Rome and entered this study. Fifty-five healthy blood donors were used as controls. None of them showed any abnormalities concerning basic laboratory tests and no detectable infection was observed. Either patients or healthy blood donors were admitted after giving informed consent.

The patient groups, whose main clinical features are reported in Table 1, included 71 monoclonal gammapathies of undetermined significance (MGUS) and 77 multiple myeloma (MM) diagnosed and staged according to Durie & Salmon criteria [26]. MM patients were classified as stage I or II when analysed at the onset of the disease and were treated with conventional therapeutic regimens including melphalan (0.25 mg/Kg body weight/day) and prednisone (2 mg/Kg body weight) for 4 consecutive days. The course was repeated at every 6^th ^week until tumour progression). The response was defined as minor response when the serum M-protein had decreased by > 25% but < 50% or the urinary BJ had decreased by >50% but not to < 0.2 g in 24 h. The non response group was defined by serum M-protein levels that had decreased to < 25% or by urine BJ protein levels that had decreased to < 50% of initial levels. Intermediate situations were categorized as a no change disease.

**Table 1:** Main characteristics of MGUS, MM patients and healthy controls

Group (n)	MGUS (71)	MM (77)	Control (55)
Gender			
Male	38	49	28
Female	33	28	27
Age (y)			
	65.9 ± 10.5	66.7 ± 10.7	59.6 ± 14.5
Isotype (H)			
IgG	62	48	--
IgA	3	28	--
IgM	6	--	--
IgD	--	1	--
Isotype (L)			
K	38	54	--
λ	33	23	--
s-M Protein (g/L)			
	9.42 ± 4.61	25.8 ± 10.7	--
Bence Jones			
Yes	41	63	--
No	30	14	--
Clinical stage (*)			
	--	I – II	--

Age is given as mean ± SD. MGUS vs MM: p = 0.11; MGUS vs CTR: p = 0.005; MM vs CTR: p = 0.0001; Gender: MGUS vs MM: p = 0.30; MGUS vs CTR: p = 0.91; MM vs CTR: p = 0.21. s-M Protein concentration is expressed as mean ± SD. MGUS vs MM: p = 0.0001 (*) according to the Durie & Salmon criteria [26].

**Figure 1:**
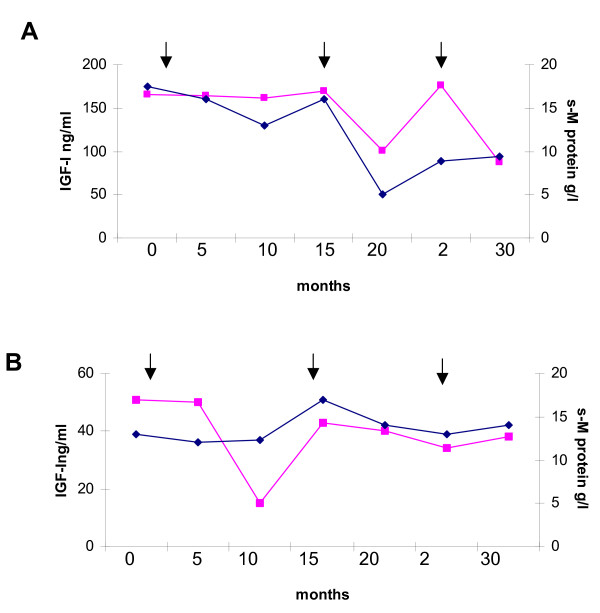
**Serial measurements of IGF-1 and serum M-Protein (s-MP) from diagnosis (0) to last follow-up before death in two MM patients**. Serum MP concentrations were derived from medical records. The first arrow indicates when MP treatment started, according to the protocol described in "Methods; the others two arrows indicate the repetition of new cycles of therapy due to disease progression. [symbols: cube = IGF-I; diamond = s-M protein].

Some of the myeloma patients, selected for having at least 6 subsequent determinations and from whom venous samples had been drawn at regular intervals starting from diagnosis, were included for a detailed analysis of the IGF-I changes during the clinical course of the disease (about 2.5 years). Two representative examples are shown in Figure 1

### Cytokine measurements

The detection of serum cytokines was performed on peripheral blood samples processed within 1 h after venipuncture by centrifugation (1500 gfor 10 min) Serum samples were collected from MGUS and MM patients as well as from 55 healthy blood donors and were stored at -70°C until testing. The angiogenic factors (VEGF and bFGF) were measured with a quantitative ELISA (Quantikine™ and Quantikine^®^; R&D Systems, Minneapolis, MN, USA), according to the manufacturer's instructions and expressed as pg/ml. All determinations were performed in duplicate in only one analytical set; the ELISA test sensitivity, expressed as mean minimal detectable dose (MDD), was < 5 pg/ml for VEGF and 3 pg/ml for bFGF. Coefficients of variation (CV) for the different cytokines obtained repeating 5 times the same samples did not exceed 15%. When necessary, samples with levels higher than the maximum standard of the calibration curve were repeated after dilution. The inter-assay CV reported by the manufacturer varies from 6.2% to 8.8% for VEGF and 7.4% to 9.1% for bFGF. The intra-assay CV varies from 5.1% to 6.7% for VEGF and 3% to 9.7% for bFGF. In order to avoid potential platelet interference with the VEGF concentration, for each patient or control subject the serum values were corrected for their relative platelet counts.

IGF-I concentration was determined as serum immunoreactivity using a quantitative sandwich enzyme immunoassay (ELISA) technique (Quantikine^® ^R&D Systems, Minneapolis, MN, USA) according to the manufacturer's instructions and expressed as ng/mL. Test sensitivity of IGF-I was 0.026 ng/ml while the inter-assay CV reported by the manufacturer for IGF-I vary from 7.5% to 8.1% and the intra-assay CV from 3.5% to 4.3%.

### DNA isolation

DNA was extracted from bone marrow aspirates using the MICRO-GENO DNA kit (AB Analitica, Padoa, Italy) according to the manufacturer's instructions. The quality of isolated DNA was analyzed through a 1% agarose gel electrophoresis.

### RFLP-PCR assay

Mutations at K-*ras *codon 12 (G**G**T→G**C**T) were detected from all samples by an "enriched" restriction fragment length polymorphism-polymerase chain reaction (RFLP-PCR) assay according to Kahn SM et al. [27], as previously described [28].

### Statistical analysis

This report primarily employed univariate analysis of the data by means of non parametric tests (Mann and Whitman or Kruskall Wallis variance analysis for quantitative and corrected X square or Fisher's exact test for categorical data).

Besides univariate analysis, a multivariate logistic regression analysis was also performed and the significances were adjusted for age and gender. This logistic regression analysis employed as end point the four variables subdivided into two groups of subjects exceeding or not the cut off value (i.e. the median value of the relative controls). The multivariate logistic regression analysis has been applied by using the SPSS version 6.0 for Microsoft Windows 95/98. This model applies the stepwise logistic regression ("SPSS backward LR method"). A p < 0.05 cut off has been employed for the significance evaluation.

## Results

### Clinical characteristics of the subjects studied

To analyze the basal characteristics of the subjects studied in this report (Table 1), we have tabulated the data concerning the main clinical features subdivided into three groups, namely: 55 healthy blood donors, 71 MGUS and 77 MM. No significant variations were registered for the gender in the three comparisons, while age significantly differed when control subjects were compared with MGUS or MM.

### Serum distributions of the four cytokines in Controls, MGUS and MM

The median serum levels and ranges of three cytokine: IGF-I, bFGF and VEGF, compared with those of healthy controls were reported in Table 2. The serum levels of IGF-I were significantly and sequentially reduced from controls to MGUS and from MGUS to MM. The significances between these three groups were always < 0.0001. In addition, these significances were more pronounced than those observed for bFGF and VEGF. A multivariate logistic regression analysis showed that the significances observed for IGF-I concentrations in the three groups were independent of age and gender and the relative p was 0.01.

**Table 2:** Serum levels of IGF-I, betaFGF and VEGF in Control, MGUS and MM

Group	N°	IGF-I ng/ml	B-FGF pg/ml	VEGF pg/ml
Controls	55	135.5 (65–279)	1.62 (1.04–2.15)	1.25 (0.15–1.95)
MGUS	71	111.3 (10–215.8)	2.08 (0.04–8.19)	1.12 (0.15–5.90)
MM	77	78 (16–352)	2.37 (0.04–82.7)	1.37 (0.3–18.3)
P1		<.0001	0.01	0.19
P2	--	<.0001	.001	.57
P3	--	<.0001	.27	.14
P4	--	<.0001	.02	.14

A statistical analysis has been performed both on the three groups together and on the different couple of groups.

Cytokine levels are given as median (range). P1 = univariate analysis, Kruskall-Wallis test on the three groups. P2 = univariate analysis, Mann Whitney test on Controls vs MGUS. P3 = univariate analysis, Mann Whitney test on MGUS vs MM. P4 = univariate analysis, Mann Whitney test on Controls vs MM.

The IGF-I behaviour has been also confirmed by logistic regression analysis after data correction for age and gender, as described in the text.

Also bFGF presented significantly different serum values among the three groups. In particular, there was a statistically significant difference (p = 0.001) between the controls and the MGUS patients, in which higher values were observed. A similar difference was registered between the controls and the MM patients (p = 0.02), while, in contrast, MGUS and MM showed similar results (p = 0.27). The multivariate analysis, corrected for age and gender, did not reach a statistical significance (p = 0.9).

VEGF, finally, did not show significant variations in the four comparisons (p at least > 0.14) and the multivariate analysis, performed as above, was also not significant (p = 0.08). A correlation matrix using the values of the four variables in MGUS or MM groups only resulted significant for VEGF vs b FGF (r = 0.37, p = 0.002) in MGUS patients.

### K-*ras *mutations in the MGUS and MM patients

Since it is known that gene alterations may be linked with cytokine modulation, we analyzed the incidence (%) of K-*ras *mutations in MGUS and MM subjects, due to the emerging role of this gene in plasma cell dyscrasia pathogenesis [29,30]. Mutations at K-*ras *codon 12 were analyzed on genomic DNA isolated from bone marrow cell specimens of the two groups of patients. Due to the presence of low-molecular weight fragments of isolated DNA, 66 out of a total of 71 MGUS samples and 73 out of a total of 77 MM samples were considered suitable for genetic analyses.

The K-*ras *gene mutations were present in only one (1,5%) MGUS subject and in twenty (27,4%) MM ones. As expected, none of the control specimens analyzed manifested gene alterations (Table 3). In fact, it was observed a highly significant (p < 0.0001) difference between the controls and MM or between MGUS and MM, while no significance was found between controls and MGUS groups (p = 0.95) by means of a two by two comparison of the three groups (controls, MGUS and MM) concerning the distribution of K-*ras *gene mutation,

**Table 3:** K-*ras *gene status and response to therapy

Group	K12-*ras *gene mutation/total (%)	Positive therapy response (%)		P Value
		Mutant	Wild type	
Controls	0/75 (0)	__	__	__
MGUS	1/66 (1.5)	__	__	__
MM	20/73 (27.4)	26.9	58.3	0.01

Statistical significance for K12-ras gene mutation: Control vs MGUS p = 0.95, Control vs MM p = 0.0001, MGUS vs MM p < 0.0001, Positive therapy response: minor response and no change disease **(see Methods)**.

Interestingly, significant increases (P = 0.02) of serum bFGF levels were observed in patients showing K-*ras *gene mutation (median = 4.6 pg/ml; range = 1.2–19.6 pg/ml) as compared with those in which the gene was in the wild type form (median = 2.2 pg/ml; range = 1.0–20.8 pg/ml). No statistically significant differences between K-*ras *gene status and serum factor concentrations were found for IGF-I or VEGF.

### MM response to Melphalan therapy

Seventy-three MM patients showing or not K-*ras *gene mutations were analyzed for their response to therapy. As shown in Table 3, the presence of K-*ras *mutations was significantly associated with a lower response to Melphalan as compared with the wild type K-*ras *subjects (p = 0.015). A statistically not significant trend (p = 0.07) was also observed for the serum bFGF concentrations when comparing responders (mean = 1.9 pg/ml; range = 1.2–20.8 pg/ml) with non responders (mean = 3.8 pg/ml; range = 1.3–19.6 pg/ml).

In an attempt to find a link between the response to therapy (yes/not), K-*ras *gene status (mutant/wild type) and the cytokine level (greater or lower than cut-off), we could only confirm the strong influence of K-*ras *gene status rather than the level of the four different cytokines in determining the therapy response of MM patients (data not shown).

### Monitoring of two MM patients for Monoclonal component concentration and serum IGF-1 levels

Several patients were followed up during therapy. Figure 1 shows two of them presenting at least six/seven observation times in which consecutive serum samples from the time of diagnosis until death were analyzed. The first patient (panel A) had a high serum IGF-I (165 ng/ml) level at diagnosis. He showed a minor response to treatment for a least 15 months, with a 26% fall in serum M-protein concentration and a concomitant slight reduction of IGF-I amounts. Then new cycles of therapy were administered because of tumour progression. The patient responded to treatment for about 10 months (s-M protein = 6 g/l; serum IGF-I level = 101 ng/ml), then relapsed again. New treatment was then initiated, but the patient died after about 5 months.

The second patient (panel B), which had a normal serum IGF-I (51 ng/ml) concentration at diagnosis, did not respond to treatment and with the exception of an IGF-I reduction observed 10 months after starting therapy, he showed only slight modifications of both serum variables during the course of disease.

## Discussion and conclusion

MM evolution has been shown to be strongly conditioned by angiogenic mechanisms in terms of growth and therapy sensitivity. Several authors tried to explain how angiogenic cytokines [4,31] may work influencing the MM cells; consequently, in the recent years, the presence and quantity of several angiogenic factors, their inducers and their signalling mediators have been documented in an effort to explore the possibility to use them as diagnostic, monitoring or prognostic markers of disease evolution and therapy sensitivity.

Despite this bulk of information, clear indications have not been completely gained and some different contrasting results have been published [4,8,9,32-34]. In general, angiogenic mediators (VEGF, basic FGF, TGF-beta1, TNF-alpha) have been found to be increased in MM patients and often significantly correlated each to the others [8]. Sometimes, they were also stage related, although not all the reports were consistent in this field. Angiogenic factors also show different behaviours under treatment. Interestingly, while conventional therapy (melphalan plus prednisolone) reduced the serum concentrations of these factors [35], an anti-angiogenic therapy based on thalidomide plus dexamethasone was accompanied by increase of the same factors in the responder subjects [4,34].

Another molecule involved in MM biology is IGF-I, a mediator with cytokine (locally)/hormone (in the general circulation) activity [36], known to be a growth promoter for several tumours, including MM, acting through its anti-apoptotic/proliferative [16,19,37] effects and interaction with angiogenic factors, such as the anti-proliferative TGF-beta1 [38].

Surprisingly, serum data regarding IGF-I and MM are very scarce and partially contrasting [39,40] although IGF-I is suspected to be able to transform MGUS in MM [41]. Previous data on B-cell chronic lymphocytic leukaemia have found a clear reduction of IGF-I in sera as compared with controls [42], even though the studies were initially expected to exibit increased concentrations, due to the tumourigenic activity of IGF-I. This is not so surprising in that the IGF-I determinations were obtained from sera of subjects already affected with MM.

It is known that all cancers, including MM, possess a more or less strong inflammatory component (in particular the proinflammatory cytokines IL-6 and TNF-alpha are produced by myeloma cells) and that inflammation is associated with reduced IGF-I synthesis [43]. Data from literature showed that IGF-I molecules may be modulated by SOCS genes, after their activation by proinflammatory cytokines [37].

The present study clearly shows that the serum IGF-I concentrations significantly decreased from healthy blood donors to MGUS and to MM patients, a finding not previously described. This result was also independent of age (significantly lower in controls) and sex, as confirmed by multivariate regression analysis, both in all subjects and when only IgG MM patients were considered (data not shown). A similar analysis, obtained separately in male or female patients, confirmed our findings (data not shown), indicating that gender was not the cause of the differences previously described. These findings open the possibility that IGF-I molecule might be further studied as a monitoring marker to follow the patients over time by specific trials.

A previous study by Standal and coworkers [39], failed to observe significant differences between MM and controls. Such divergence may depend on some patient characteristics. For example, Standal selected only patients with 69% of advanced tumour stages (III), while our patients were prevalently of tumour stages I and II. As previously mentioned, chronic B cell leukaemia showed data similar to those reported in our paper [42].

Opposite to IGF-I was the behaviour of VEGF and bFGF, whose concentrations were increased in MM sera as compared with control samples. VEGF and b-FGF serum concentrations were highly correlated (P = 0.002), confirming the results previously published by other authors [8].

Another variable considered in this study was the K-*ras *gene whose mutation was significantly associated with the malignancy [29,30], while no significant difference was observed between controls and MGUS. K-*ras *gene alteration has previously been associated with the modulation of different biological agents, including IGF-I [23,24,44,45]. As reported for solid tumours [47], we found significant increases of serum bFGF concentrations in MM patients eliciting K-*ras *gene activation. Moreover, the same K12-*ras *mutation was significantly associated with increased resistance to the therapy (Table 3). A trend in lower serum bFGF levels was observed when responders MM patients were compared with the non responder ones. When K12-*ras *mutation and the levels of the 3 cytokines under or above cut offs were combined, no significant differences were found in the different subgroups (data not shown). Therefore, therapy effect was only dependent on K-*ras *mutation and not on cytokine levels.

Considering the results of the present study, we tried to evaluate the possibility that IGF-I might be used as monitoring marker. Therefore, we show two representative examples of MM patients followed during subsequent courses of therapy and whose disease behaviour was related to the monoclonal component concentrate on and serum IGF-I levels over time.

These results show that, generally, serum IGF-I concentrations behave similarly to the monoclonal component. Some of the divergences observed may be explained by the fact that various eveniences may influence the serum IGF-I levels: age and gender [47], inflammatory processes [48], other concomitant diseases [49,50], endocrine diseases [47], nutrition [47], drug administration and liver toxicity. Furthermore, melphalan therapy, which is hepatotoxic and therefore should reduce IGF-I synthesis, has been reported to increase IGF-I molecules after the 4^th ^course [40], possibly when it was effective in restoring the peripheral blood IGF-I amounts. It is also possible to speculate that the cytotoxic effect of therapy should release a great amount of endocellular molecules from necrotic cells with induction of inflammatory processes and IGF-I drop.

In conclusion, as previously reported for other neoplastic diseases [42,51], serum IGF-I concentrations are clearly reduced in case of open disease. Therefore, a clinical use of serum determinations of this molecule should be made very carefully since this substance does not show a clear specificity for MM. A possible role of IGF-I as putative monitoring marker of malignant disease seems to emerge by our study, even though specific clinical trials need to be planned and the possible interference of other factors in serum determinations should be considered.

## Competing interests

All contributing authors declare that no actual or potential conflicts of interest do exist.

## Authors' contributions

CG and FA conceived of the study, discussed the results and wrote the manuscript. GV participated in the design and results discussion of the ELISA experiments. RV carried out PCR experiments on K-ras gene mutation and ELISA assays., GV participated in the revision of the manuscript, DG and IS performed statistical analysis. FP collected the biological samples and patient's clinical data. MCP participated in the study design and in the discussion of clinical data. EC discussed the results and helped to draft the manuscript.
